# First Reported Case of Hemopericardium Related to Dabigatran Use Reversed by New Antidote Idarucizumab

**DOI:** 10.1155/2017/6458636

**Published:** 2017-06-14

**Authors:** Steven Song, Joselle Cook, Clive Goulbourne, Matthew Meade, Louis Salciccioli, Jason Lazar

**Affiliations:** ^1^Department of Medicine, SUNY Downstate Medical Center, Brooklyn, NY 11203, USA; ^2^Division of Cardiovascular Medicine, SUNY Downstate Medical Center, Brooklyn, NY 11203, USA

## Abstract

Dabigatran, the first novel oral anticoagulant (NOAC) with a reversal agent, heralded a paradigm shift in the treatment of nonvalvular atrial fibrillation. The potential for life-threatening hemorrhagic events with the use of NOACs has been highly debated since the effectiveness of reversal agents such as idarucizumab is based primarily on pharmacologic data. It is known that cancer patients are at an increased risk of bleeding with anticoagulation, though specific studies demonstrating the risks or efficacy of NOACs in this population are lacking. We provide the first report of hemopericardium resulting in multiorgan failure related to dabigatran use that was successfully reversed by idarucizumab in a man with prostate cancer on chemotherapy.

## 1. Introduction

Dabigatran, the first novel oral anticoagulant (NOAC) with a neutralizing reversal agent, ushered in a paradigm shift for the treatment of nonvalvular atrial fibrillation [1]. The inherent bleeding risk and the potential for life-threatening hemorrhagic events with the use of dabigatran and other NOACs have been a highly debated topic of interest. Until very recently, there was no approved antidote for these drugs, whereas reversal of warfarin toxicity can be accomplished with fresh frozen plasma or 4-factor prothrombin complex concentrate [2]. Reports of atypical and unexpected hemorrhagic events in association with NOACs have surfaced, warranting the reassessment of the risk-benefit of these drugs along with the reliability of published clinical trials in different patient subsets [3, 4]. This case describes a spontaneous hemopericardium related to the use of dabigatran presenting as multiorgan failure and successful reversal by idarucizumab.

## 2. Case Presentation

An 84-year-old man with atrial fibrillation on dabigatran, and castrate resistant prostate cancer with bone metastases, was transferred to our medical center for further management of neutropenic fever and acute kidney injury. The patient stated he started feeling progressively weaker immediately after receiving his last dose of chemotherapy (enzalutamide and docetaxel) two weeks ago. The patient was compliant with his dabigatran (150 mg twice daily), last dose taken a few hours prior to presentation. Upon admission, the patient was hemodynamically stable but febrile to 101°F. Physical exam revealed significant bilateral lower extremity edema. Labs were notable for neutropenia; white blood cell count was 1.1 × 10^9^ cells/L with an absolute neutrophil count of 400 cells/*μ*L. Renal injury was evident with blood urea nitrogen of 53 mg/dL and creatinine of 1.7 mg/dL, increased from his baseline of 14 mg/dL and 0.7 mg/dL, respectively, 10 days priorly. Serum potassium was elevated at 5.4 mEq/L. Activated partial thromboplastin time (aPTT) was prolonged at 47.7 seconds. Liver enzyme tests were normal. Electrocardiogram showed rate-controlled atrial fibrillation with no acute changes. Chest X-ray showed probable infiltrates suspicious for pneumonia. Intravenous hydration was initiated and piperacillin/tazobactam administered for empiric coverage of his neutropenia and presumptive pneumonia. His home dose dabigatran was continued and he received his first dose the morning after admission.

**Figure 1:**
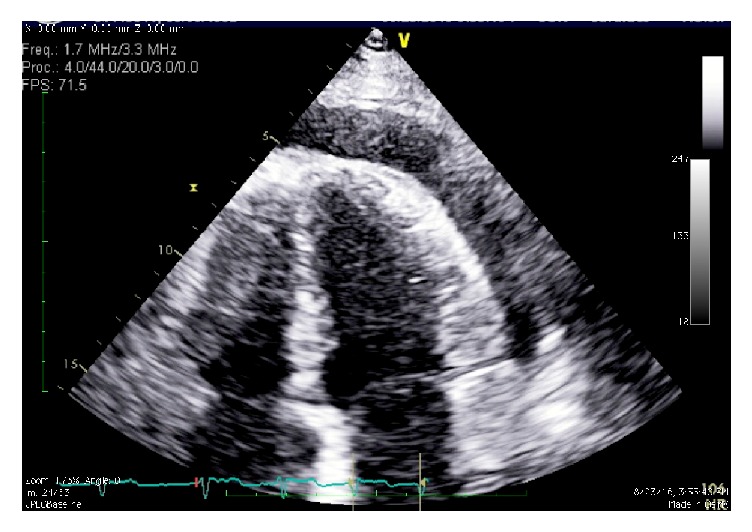
Two-dimensional transthoracic echocardiogram showing a large pericardial effusion in the apical view.

**Figure 2:**
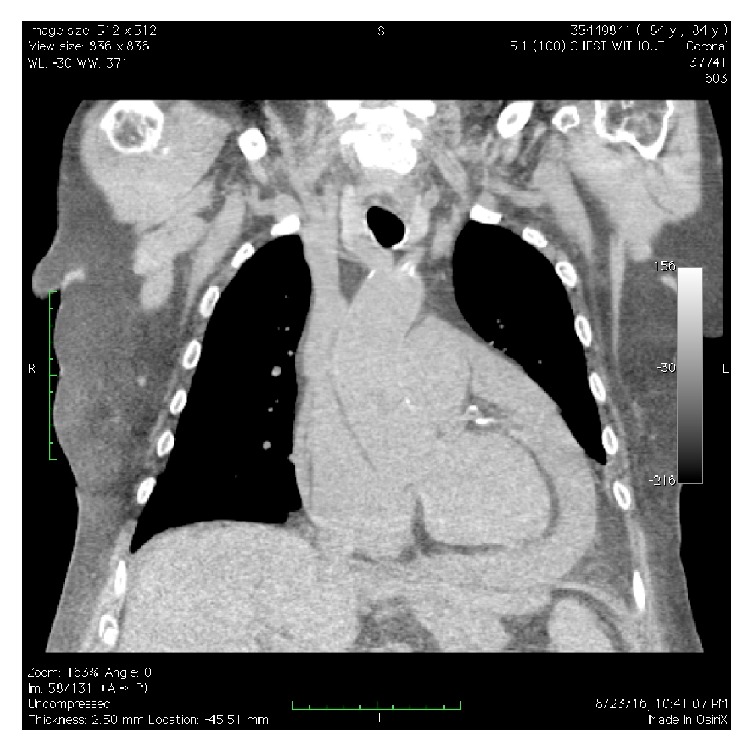
Computerized tomography (CT) of the chest showing new pericardial effusion and small bilateral pleural effusions with adjacent atelectasis when compared to the CT of the chest performed 21 days priorly.

Within 24 hours, the patient was notably lethargic and oliguric. Labs demonstrated deterioration in renal function, worsening hyperkalemia, and acute elevation in transaminases (aspartate aminotransferase 635 mg/dL, alanine transaminase 383 mg/dL). Sepsis work-up was negative. Computerized tomographic imaging of the brain and abdominal ultrasound were also unrevealing. Transthoracic echocardiography demonstrated a large pericardial effusion and decreased filling of the left ventricle and dilatation of the inferior vena cava with a decrease in inferior vena cava respiratory variation suggesting elevated right atrial pressure, indicating early cardiac tamponade (Figure 1). Computerized tomography of the chest confirmed the pericardial effusion (Figure 2). Dabigatran was immediately stopped. Cardiothoracic surgery and interventional radiology were consulted for emergent intervention, considering the patient was anticoagulated with dabigatran.

Within hours, the patient became hemodynamically unstable with further deterioration in mental status. The examination was notable for prominent jugular venous distention, pulsus paradoxus, distant heart sounds, and diffuse bilateral wheezes and crackles on auscultation. Repeat labs demonstrated evidence of multiorgan failure with metabolic acidosis (serum bicarbonate 14 mg/dL, lactate 4.9 mg/dL) and significant deterioration in hepatic and renal function (blood urea nitrogen level 80 mg/dL, creatinine 2.9 mg/dL, aspartate aminotransferase 826 mg/dL, and alanine transaminase 548 mg/dL). Electrocardiogram now revealed atrial fibrillation with rapid ventricular response and diffuse low voltage across all leads. A repeat transthoracic echocardiogram now demonstrated cardiac tamponade. The patient was emergently transferred to the intensive care unit for cardiogenic shock and multiorgan failure and subsequently started on norepinephrine for hemodynamic support. Idarucizumab was administered. Activated partial thromboplastin time (aPTT) shortly after idarucizumab administration normalized to 29.5 seconds (compared to 47.7 seconds on admission). The patient underwent successful interventional radiology guided pericardiocentesis without complication. Activated partial thromboplastin time (aPTT) the morning after pericardiocentesis remained normalized at 30.8 seconds. Initially 700 mls of grossly hemorrhagic fluid was removed, with an additional 200 mls over several days via the pericardial drain. Cytology was negative for malignant cells. Red blood cell count by volume of the pericardial fluid was greater than 50% of serum, consistent with hemopericardium.

The patient recovered to his baseline status over one week, with laboratory and clinical resolution of his multiorgan failure and no echocardiographic evidence of hemopericardium.

## 3. Discussion

NOACs pioneered a new era in revolutionizing oral anticoagulation treatment for nonvalvular atrial fibrillation and venous thromboembolic events [4, 5]. These agents have more predictable pharmacodynamic and pharmacokinetic properties than warfarin and relatively lower (but not absent) potential for interactions with drugs, herbal, and dietary constituents, which has obviated the need for routine laboratory monitoring [4]. However, other considerations are important including the need for dosage adjustments in renal impairment and avoidance in severe liver impairment. Clinical trials demonstrate fewer hemorrhagic complications and drug interactions compared to warfarin, along with a more predictable steady state level in the blood [4, 6].

Without the need for routine monitoring, physicians may be lulled into a false sense of security regarding NOACs' bleeding risks. Paradoxically, the fear of bleeding with NOACs and lack of readily available reversal agents may lead to the underutilization of anticoagulation when indicated. Increased prescribing of dabigatran has been spurred by the advent of the first specific antidote idarucizumab in 2015, indicated for use in life-threatening bleeds [7, 8]. However, the evidence clearly demonstrates that patients at high risk of bleeding with traditional anticoagulation have similar high risks with NOACs [4]. Reports of atypical and severe bleeding presentations attributable to these drugs have emerged in the midst of the current fervor for NOACs [9]. Life-threatening hemopericardium is increasingly reported as a manifestation of adverse events attributed to NOACs [3, 10, 11].

It is reported in literature that drugs which strongly affect P-glycoprotein, an ATP-dependent efflux transporter, can alter plasma concentrations of dabigatran, potentially potentiating adverse hemorrhagic events [11, 12]. The authors presume that the combination of previously administered docetaxel and enzalutamide in the index patient, both modulators of the P-glycoprotein system, in addition to severe renal impairment likely potentiated dabigatran's anticoagulant effect. This resulted in the dramatic presentation of hemopericardium [12].

Though monitoring of dabigatran activity is not routine, some measure of drug activity may be required in patients suspected to have high bleeding risks or in those with suspected dabigatran toxicity. The activated partial thromboplastin time (aPTT) is reliably elevated, though with nonlinear pharmacokinetics with dabigatran use, and may be used as a proxy or a screening test in patients suspected to have a bleeding risk [13]. Normalization of aPTT shortly after idarucizumab administration as seen in this index case suggests idarucizumab's effectiveness in the reversal of the anticoagulatory effects of dabigatran [14]. However, ecarin clotting time (ECT) and diluted thrombin time (dTT), tests which are not widely available, are more sensitive and accurate measures of dabigatran activity and idarucizumab effectiveness [14, 15].

Consideration of patients' comorbidities and drug interactions is crucial in anticipating lowered thresholds for life-threatening bleeds for patients on NOACs as oncology patients already have a significantly increased bleeding risk with NOACs compared to the general population [4, 12, 16]. Furthermore, in addition to basic consideration for drug interactions between the chemotherapy and the NOAC, review of nephrotoxic, hepatotoxic, and bone marrow suppressive potential of the chemotherapy that may potentiate toxic levels of the NOAC is prudent. Until definitive practice patterns are established, NOACs should be cautiously used in the cancer population [12, 16]. Elevated aPTT levels, though suggestive, are not absolute in confirming supratherapeutic dabigatran levels. The authors hope this case sensitizes clinicians to utilizing and ordering ECT and dTT should there be a high suspicion for supratherapeutic dabigatran levels in patients at increased risk of bleeding to prevent life-threatening hemorrhagic events. Based on the presented clinical data, idarucizumab was effective in neutralizing dabigatran and was lifesaving in this presentation of massive hemopericardium [7, 8, 17, 18].
